# Supplementary material for: Design, Synthesis, and Biological Evaluation of N-Acylhydrazones and Their Activity Against Leishmania amazonensis Promastigotes
Source: Microorganisms. 2025 Jul 2;13(7):1563. doi: 10.3390/microorganisms13071563 (PMC12298860; doi:10.3390/microorganisms13071563)
Supplement: Supplementary file 1 [file microorganisms-13-01563-s001.zip › RMN amostras-20241114T155656Z-001.zip!/RMN amostras/composto 17 - PMM 81-17 1H.pdf]

PMM 81-17 - Joao Mafra
Op. Eliane

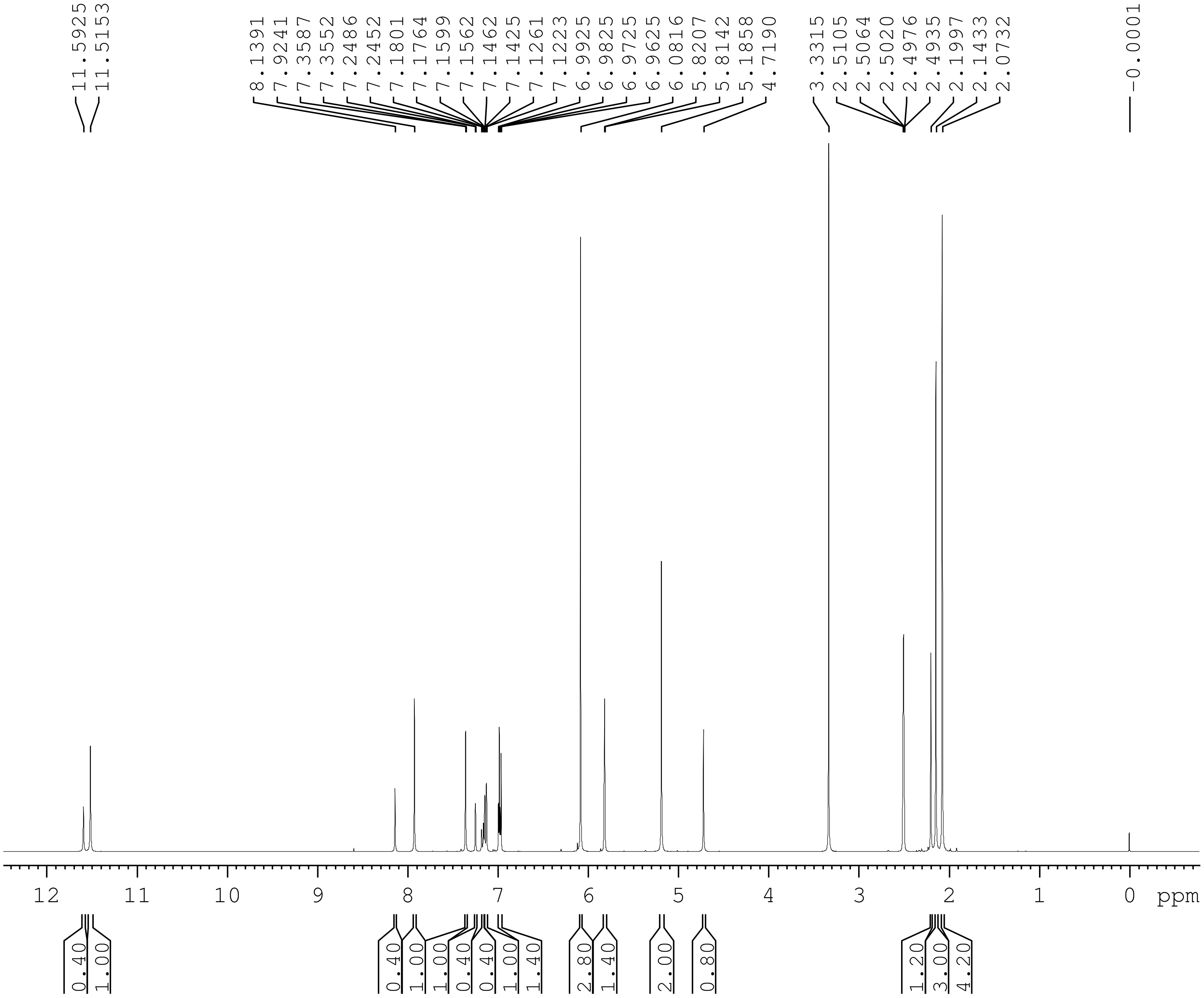

BRUKER

Current Data Parameters

NAME	si103181724
EXPNO	10
PROCNO	1

F2 - Acquisition Parameters

Date_	20180306
Time	11.17 h
INSTRUM	spect
PROBHD	Z122623_0041 (
PULPROG	zg30
TD	65536
SOLVENT	DMSO
NS	32
DS	2
SWH	8012.820 Hz
FIDRES	0.244532 Hz
AQ	4.0894465 sec
RG	116.93
DW	62.400 usec
DE	10.00 usec
TE	298.0 K
D1	1.00000000 sec
TD0	1
SFO1	400.1524711 MHz
NUC1	1H
P1	11.50 usec
PLW1	7.30000019 W

F2 - Processing parameters

SI	65536
SF	400.1500023 MHz
WDW	EM
SSB	0
LB	0.30 Hz
GB	0
PC	1.00
